# Leucocyte-derived extracellular trap formation significantly contributes to *Haemonchus contortus* larval entrapment

**DOI:** 10.1186/s13071-015-1219-1

**Published:** 2015-11-26

**Authors:** Tamara Muñoz-Caro, Mario C. Rubio R, Liliana M. R. Silva, Gerd Magdowski, Ulrich Gärtner, Tom N. McNeilly, Anja Taubert, Carlos Hermosilla

**Affiliations:** Institute of Parasitology, Justus Liebig University Giessen, Giessen, Germany; Faculty of Veterinary Medicine and Zootechny, Autonomous University of Sinaloa, Culiacán, Mexico; Institute of Anatomy and Cell Biology, Justus Liebig University, Giessen, Germany; Moredun Research Institute, Penicuik, UK

**Keywords:** *Haemonchus contortus*, Neutrophil extracellular traps, Innate immunity, ETosis

## Abstract

**Background:**

Polymorphonuclear neutrophil (PMN) and eosinophil extracellular trap (ETs) formation has recently been described as an important host effector mechanism against invading pathogens. So far, scarce evidence on metazoan-triggered ET formation has been published. We here describe for the first time *Haemonchus contortus*-triggered ETs being released by bovine PMN and ovine eosinophils in response to ensheathed and exsheathed third stage larvae (L_3_).

**Methods:**

The visualization of ETs was achieved by SEM analysis. The identification of classical ETs components was performed via fluorescence microscopy analysis. The effect of larval exsheathment and parasite integrity on ET formation was evaluated via Pico Green®- fluorescence intensities. ETs formation under acidic conditions was assessed by using media of different pH ranges. Parasite entrapment was evaluated microscopically after co-culture of PMN and L_3_. ET inhibition experiments were performed using inhibitors against NADPH oxidase, NE and MPO. Eosinophil-derived ETs were estimated via fluorescence microscopy analysis.

**Results:**

L_3_ significantly induced PMN-mediated ETs and significant parasite entrapment through ETs structures was rapidly observed after 60 min of PMN and L_3_ co-culture. Co-localization studies of PMN-derived extracellular DNA with histones (H3), neutrophil elastase (NE) and myeloperoxidase (MPO) in parasite-entrapping structures confirmed the classical characteristics of ETs. *Haemonchus contortus*-triggered ETs were significantly diminished by NADPH oxidase-, NE- and MPO-inhibition. Interestingly, different forms of ETs, i.e. aggregated (*agg*ETs), spread (*spr*ETs) and diffused (*diff*ETs) ETs, were induced by L_3_. *Agg*ETs and *spr*ETs firmly ensnared larvae in a time dependent manner. Significantly stronger *agg*ETs reactions were detected upon exposure of PMN to ensheathed larvae than to exsheathed ones. Low pH conditions as are present in the abomasum did not block ETosis and led to a moderate decrease of ETs. Eosinophil-ETs were identified extruding DNA via fluorescence staining.

**Conclusion:**

We postulate that ETs may limit the establishment of *H. contortus* within the definitive host by immobilizing the larvae and hampering them from migrating into the site of infection. Consequently, *H. contortus*-mediated ET formation might have an impact on the outcome of the disease. Finally, besides PMN-triggered ETs, we here present first indications of ETs being released by eosinophils upon *H. contortus* L_3_ exposure.

**Electronic supplementary material:**

The online version of this article (doi:10.1186/s13071-015-1219-1) contains supplementary material, which is available to authorized users.

## Background

*Haemonchus contortus* is a gastrointestinal nematode with worldwide distribution. This abomasal parasite leads to significant economic losses particularly in small ruminant livestock [1]. Haemonchosis is acquired by ingesting infective third-stage larvae (L_3_) from contaminated pasture. After exsheathment, L_3_ penetrates the abomasal glands where it develops to fourth-stage larvae (L_4_) and thereafter to dioecious haematophagous pre-adult larvae (L_5_) and adults. Adult *H. contortus* nematodes are found in the lumen of the abomasum in herbivores [2–5]; or the stomach of omnivores [6] consuming up to 0.05 ml host blood per worm daily [7], which results in hemorrhagic abomasitis (gastritis), anaemia, oedema and associated complications often leading to death of severely infected animals [8].

*H. contortus* infections in ruminants are known to elicit a Th2 type-dominated host immune response being characterized by the recruitment of large numbers of eosinophils, mast cells and globule leucocytes and to the production of locally active and circulating antibodies [9–11]. Nonetheless, little is known on the very early host innate immune responses against *H. contortus.* In this scenario, the relative inaccessibility of infective *H. contortus* L_3_ within abomasal/gastric glands for host leucocytes poses unique challenges to the innate immune system, which has evolved several specialized strategies for parasite control [12]. Parasite colonization of the host abomasum initially depends on the motility of the larvae and the parasite load. Thus, some host individuals, after sensitization via previous infections, can modify the microenvironmental conditions of this niche to expel the parasite [13]. There is evidence showing that helminths activate the alternative complement pathway binding opsonins on their surface [14]. Moreover, within the innate immune response polymorphonuclear neutrophils (PMN; [15]) and eosinophils are considered as fundamental leucocytes forming the first line of defense against metazoan nematodes and the first leucocytes to be recruited to the site of infection [16–19]. Various authors have reported that eosinophils are capable of immobilizing infective larvae of diverse species of nematodes in vitro and in vivo [9, 10, 20, 21]*.* Furthermore, incubation of *H. contortus* L_3_ with antibodies raised against HcsL_3_ antigens in the presence of ovine eosinophils resulted in significant larval killing after 24 h [20]. In addition, it has been demonstrated that eosinophils are essentially involved in the expulsion of diverse nematodes in vivo, such as *Strongyloides stercoralis* [22], *Onchocerca lienalis* [23], *Trichinella spiralis* [24] and *Trichostrongylus colubriformis* [25]. Alongside phagocytosis and oxidative burst, leucocytes are capable of triggering extracellular traps (ETs) as a novel effector mechanism. This results in the cellular release of granule proteins and chromatin upon activation that together form extracellular fibers capable of binding and killing Gram-positive and -negative bacteria and parasites [16, 26]. So far, the mechanism of ET formation has been attributed to PMN [16], mast cells [27], macrophages [28], eosinophils [29] and monocytes [30, 31] and thus appears to be a general effector mechanism of innate immune cells. Most studies on pathogen-triggered ETs have been focused on bacterial, viral and fungal infections [17, 32–34]. However, little attention has been paid to parasites as ET-inducers [26] and studies of ET induction by parasites have mainly focused on protozoans [35–40]. So far, only two helminth species, i.e. *Schistosoma japonicum* [41] and *Strongyloides stercolaris* [15], have been proven to induce NETs.

With the present work we add a new species to the group of metazoan-ET-inducers and highlight the capability of ETs to entrap this large parasite. The current data suggest *H. contortus*-mediated NET formation may influence the outcome of the infection in affected animals in vivo. Furthermore, we here provide first evidence on eosinophil-derived ET formation in response to a helminth parasite.

## Methods

### Ethic statement

All animal procedures were performed according to the Moredun Research Institute regulations and to the Justus Liebig University Animal Care Committee guidelines, approved by the Ethic Commission for Experimental Animal Studies of the State of Hesse and in accordance to the current European Animal Welfare Legislation: ART13TFEU.

### Animals and parasites

Three male Merino sheep were purchased from a local farmer at the age of five months, treated with a single dose of toltrazuril [20 mg/kg body weight (bw), Baycox® 5 %; Bayer Animal Health] and benzimidazole (10 mg/kg bw, Panacur; Bayer Animal Health). The sheep were controlled for parasitic infections via weekly coprological analyses and, when deemed parasite free, maintained under parasite-free conditions within a large animal stable equipped with a laminar flow lock entrance until experimental infection (Institute of Parasitology, Justus Liebig University, Giessen, Germany). Animals were fed with commercial starter pellet concentrates (Lämmerpellets®, Deuka). Water and sterilized hay were given *ad libitum*.

Animals were infected *per os* with 8 × 10^3^ viable ensheathed *H. contortus* L_3_ (in house strain) suspended in tap water. Following prepatency of approximately three weeks, cotton faecal collection bags were fixed to the anuses of sheep to collect faeces and were emptied each day.

The isolation of excreted *H. contortus* eggs and exogenous in vitro culture into third stage larvae were performed as previously described elsewhere [42]. Faecal samples (10–50 g) were transferred to a jar and mixed with commercially purchased sawdust until a crumbly consistency was obtained, and, if necessary, dampened with tap water. Thereafter, the jars were capped and incubated at 27–28 °C for 7–8 days. After incubation, tap water was added to the culture until the jar was filled up to the brim, the jar was turned upside down on a petri dish. Then, 10–20 ml of tap water was added into the petri dish and the jars were incubated overnight at room temperature (RT). Thereafter, the fluid containing *H. contortus* ensheathed L_3_ was collected, transferred to a conical tube (Greiner) and the L_3_ were sedimented at unit gravity (at least 30 min, RT). Afterwards, the supernatant was discarded; the L_3_ of *H. contortus* were counted, suspended in sterile PBS and stored at 4 °C until further use. L_3_-related ET experiments were performed within 4 weeks after parasite collection in order to prevent morphological alterations or death of the larvae.

### Exsheathment of *Haemonchus contortus* L_3_

For exsheathment, vital ensheathed L_3_ (1500 larvae in 5 ml tap water) were exposed to sodium hypochlorite solution (0.3 % *v*/*v*; 5 min, RT, Merck). Active larval exsheathment was observed under an inverted DMIRB® microscope (Leica). When at least 80 % of the *H. contortus* L_3_ had exsheathed, the larvae were washed in sterile PBS (400 × *g*, 3 × 5 min) and kept at 4 °C for further use within the same day.

### Isolation of bovine PMN and ovine eosinophils

For PMN isolation, healthy adult female Holstein-Frisian cows (*n* = 3) were bled by puncture of the jugular vein. Heparinized blood was diluted under sterile conditions in an equal volume of sterile phosphate buffered saline (PBS) containing 0.02 % EDTA (Sigma-Aldrich). The mixture was layered on Biocoll® separating solution (Biochrom AG) and centrifuged at 800 × *g* for 45 min. After removal of plasma, lymphocytes and monocytes in the upper layers of the gradient, the cells were re-suspended in 25 ml sterile distilled water to lyse erythrocytes. Osmolarity was immediately readjusted by adding 10x sterile Hank’s Balanced Salt Solution (HBSS, Biochrom AG) and the cells were pelleted (400 × *g*, 10 min). Thereafter, the cells were washed twice (400 × *g*, 10 min, 4 °C) in complete RPMI 1640 medium without phenol red (Sigma-Aldrich). The cells were counted in a Neubauer haemocytometer chamber and incubated at 37 °C and 5 % CO_2_ atmosphere for at least 30 min before use. The PMN-enriched samples had > 90 % purity.

For ovine eosinophil isolation, heparinized blood (25 ml) was collected from Texel-cross lambs (*n* = 3), with patent *H. contortus* infection, diluted 1:2 with sterile PBS- containing 0.05 % EDTA (Sigma-Aldrich) and layered on top of a 70 % Percoll gradient (25 ml, Sigma-Aldrich). The sample was centrifuged (20 min, 400 × *g*, 4 °C). Within the gradient, the eosinophil-enriched layer appeared in between the peripheral blood mononuclear cell- (PBMC) and the PMN-rich layer. Eosinophils were withdrawn by aspiration in a sterile Pasteur pipette, re-suspended in 25 ml PBS-EDTA and washed in PBS (400 × *g,* 2 × 5 min, 4 °C). The cells were re-suspended in culture medium [RPMI 1640, 10 % heat inactivated foetal calf serum (hiFCS), 2 mM L-glutamine, 100 U/ml penicillin, 100 μg/ml streptomycin and 50 μM 2-mercaptoethanol, all Sigma-Aldrich]. Eosinophil purity was determined by light microscopy of cells stained with Diff-Quick and haematoxylin-eosin staining after cyto-centrifugation onto glass slides. Eosinophil counting was performed in a Neubauer haemocytometer chamber. Eosinophils were incubated at 37 °C and 5 % CO_2_ atmosphere for at least 30 min before use. The viability of eosinophils was tested by using the trypan blue exclusion test (Sigma-Aldrich). Eosinophil-enriched samples had 30 % eosinophils.

### Scanning electron microscopy (SEM)

Bovine PMN (*n* = 3, 5 × 10^5^) were co-cultured with vital ensheathed L_3_ of *H. contortus* (100 larvae/sample) on poly-_L_-lysine (Sigma-Aldrich) pre-coated coverslips (60 min, 37 °C). After incubation, the samples were fixed in 2.5 % glutaraldehyde (60 min, RT, Merck), post-fixed in 1 % osmium tetroxide (Merck), washed in distilled water, dehydrated, critical point dried by CO_2_-treatment and spayed with gold. Thereafter, samples were examined with a Philips XL30 scanning electron microscope at the Institute of Anatomy and Cell Biology, Justus Liebig University Giessen, Germany.

### Visualization of NETs and detection of histones, neutrophil elastase (NE) and myeloperoxidase (MPO) in *Haemonchus contortus*-induced NETs

Bovine PMN (*n* = 3, 5 × 10^5^) were co-cultured with vital ensheathed *H. contortus* L_3_ (100 larvae/sample) on poly-_L_-lysine-treated coverslips (60 min, 37 °C). Thereafter, the samples were fixed (overnight, 4 % paraformaldehyde, on ice, Merck) and stored at 4 °C until further use. The experiments were performed in duplicates. In each set of data, 2 coverslips were used per condition and the entire coverslip was further analysed. NET structures were visualized by staining extracellular nucleic acid with Sytox Orange® as previously recorded [43–45]. For the detection of histones, MPO and NE within NET structures the following specific antibodies were used: anti-histone H1, H2A/H2B, H3, H4 monoclonal antibody [mouse clone H11-4, 1:1000, 1 h, RT, Merck Millipore], anti-MPO antibodies (rabbit polyclonal anti-MPO, Alexa Fluor 488, 1:200, 24 h, RT, ABIN906866, antibodies-online.com) and anti-NE antibodies (rabbit polyclonal anti human NE, 1:200, 24 h, RT, AB68672, Abcam). Prior to antibody exposure, the samples were blocked with bovine serum albumin (30 min, BSA 2 %, Sigma-Aldrich). Following antibody exposure, the samples were washed twice with PBS and incubated in respective secondary antibody solutions [Alexa Fluor 488 goat anti-mouse IgG and Alexa Fluor 488 goat anti-rabbit IgG (both from Life Technologies) 30 min, 1:500, RT]. Finally, the samples were stained with Sytox Orange® (S-11368, Invitrogen, 1:1000, 15 min in the dark), washed with PBS and mounted in anti-fading buffer (Mowiol®; Sigma-Aldrich). The visualization of NETs based on co-localized extracellular DNA staining and histone-, MPO- and NE-derived signals was achieved by using an inverted Olympus IX81® fluorescence microscope.

### Visualization of eosinophil-derived *Haemonchus contortus*-induced ETs

Co-culture of ovine eosinophils (*n* = 3, 1 × 10^6^) with either ensheathed or exsheathed *H. contortus* L_3_ (150 larvae/sample each) was carried out on poly-_L_-lysine-treated coverslips (60 min, 37 °C). After the incubation period, the samples were fixed (overnight, 4 % paraformaldehyde, on ice). Eosinophilic ET structures were visualized by staining extracellular DNA with Pico Green® (Life Technologies, 1:200 diluted in 10 mM Tris/1 mM EDTA) according to [45]. Moreover, eosinophilic granules were labelled with alcoholic Eosin Y® solution (10 s, RT, Sigma-Aldrich) and thereafter washed twice with sterile PBS. The cells were then mounted in ProLong® anti-fading reagents (Life Technologies). The visualization of eosinophil-derived ETs was achieved using an inverted Olympus IX8® phase contrast/fluorescence microscope.

### Quantification of *Haemonchus contortus-*induced NETs

Bovine PMN (*n* = 3; 5 × 10^5^ cells/200 μl) were exposed either to vital ensheathed larvae (40 larvae/sample) or heat-inactivated (60 °C, 60 min) larvae (40 larvae/sample) in RPMI 1640 medium (1 % PS, without phenol red) for 60 min at 37 °C in 96-well flat-bottom plates (Nunc). Thereafter, micrococcal nuclease was added (5 U/well, New England Biolabs) and incubated for 15 min at 37 °C. Then the samples were centrifuged (250 × *g*, 5 min) and the supernatants (100 μl/well) were transferred to a 96-well flat-bottom plate (Nunc). The samples were stained by Pico Green® (50 μl/well, 1:200 in 10 mM Tris/1 mM EDTA) and NET formation was quantified by spectrofluorometric analysis at an excitation wavelength of 484 nm and emission wavelength 520 nm using an automated plate monochrome reader (Varioskan Flash®; Thermo Scientific). For negative controls, PMN in plain medium were used. Stimulation of PMN with zymosan (1 mg/ml, Invitrogen) served as positive control. For the quantification of the different types of NETs, bovine PMN (*n* = 3, 5 × 10^5^) were seeded on poly-_L_-lysine pre-coated coverslips and exposed to *H. contortus* L_3_ (20 larvae/sample) in 300 μl RPMI medium (1 % PS, without phenol red) for 30 min and 1 h at 37 °C. Thereafter NET formation was detected via fluorescence microscopy analysis using an anti-histone H1, H2A/H2B, H3, H4 monoclonal antibody [mouse clone H11-4, 1:1000, 1 h, RT] jointly with a secondary antibody (Alexa Fluor® 488 goat anti-mouse IgG). Moreover, for staining extracellular DNA, Sytox Orange® (S-11368, Invitrogen, 1:1000, 15 min in the dark) was used.

### Entrapment assay of *Haemoncus contortus* L_3_

Bovine PMN (*n* = 3, 5 × 10^5^) were seeded on poly-_L_-lysine pre-coated coverslips and exposed to ensheathed *H. contortus* L_3_ (100 larvae/sample) in 300 μl RPMI medium (1 % PS, without phenol red, Sigma-Aldrich) for 30 and 60 min at 37 °C. Thereafter, the coverslips were fixed (4 % paraformaldehyde, overnight on ice) and PMN-entrapped larvae were counted by using an inverted DMIRB® phase-contrast microscope (Leica). Larvae were considered as entrapped when *agg*NET or *spr*NET were in contact with the larvae. The data are expressed as percentage of entrapped L_3_ relative to the total amount of *H. contortus* L_3_.

### NET-associated larvicidal assay

Bovine PMN (*n* = 3, 5 × 10^5^) were seeded on poly-_L_-lysine pre-coated coverslips and exposed to ensheathed *H. contortus* L_3_ (50 larvae/sample) in 300 μl RPMI medium (1 % PS, without phenol red) for 24, 36 and 48 h at 37 °C. Thereafter, DNase I (Sigma Aldrich) was added to the coverslips to resolve NET structures and larval survival was determined microscopically based on the presence or absence of larval motility. In addition, parasite viability was evaluated by using the trypan blue exclusion test (1:10 dilution, Sigma-Aldrich). For reference samples, the equal number of non-PMN-exposed larvae was used.

### Identification and quantification of aggregated NETs (*agg*NETs) induced by *Haemonchus contortus* L_3_

Bovine PMN (*n* = 3, 5 × 10^5^) were seeded on poly-_L_-lysine pre-coated coverslips and exposed to both, ensheathed and exsheathed *H. contortus* L_3_ (50 larvae/sample) in 300 μl RPMI medium (1 % PS, without phenol red) for 1 h at 37 °C. Thereafter, the samples were fixed (4 % paraformaldehyde, overnight, on ice). Each sample was analysed microscopically using an inverted phase-contrast microscope (DMIRB®, Leica). *Agg*NETs were defined as clusters of NET-like structures with a “ball of yarn” morphology (see Fig. 2f red arrow) being larger than 50 μm in diameter. Within each sample, all structures with the described characteristics were counted. For negative controls, PMN in plain medium was used. Stimulation of PMN with zymosan (1 mg/ml) served as positive controls as described elsewhere [45].

### NET inhibition assays

For NET inhibition assays the following inhibitors were used according to [46] and [39]: the NADPH oxidase inhibitor diphenylene iodonium (DPI, 10 μM, Sigma-Aldrich), the MPO inhibitor 4-Aminobenzoic acid hydrazide (ABAH, 100 μM, Calbiochem) and the NE inhibitor Suc-Ala-Ala-Pro-Val chloromethyl ketone (CMK, 1 mM, Sigma-Aldrich). PMN (*n* = 3, 5 × 10^5^) were pre-incubated with respective inhibitors (30 min, 37 °C, 5 % CO_2_) prior to exposure to vital ensheathed L_3_ (80 larvae/sample, 60 min). For no-inhibitor controls, non-treated PMN were exposed to larvae. For negative controls, PMN in plain medium were used. Stimulation of PMN with zymosan (1 mg/ml) served as positive control. NET formation was quantified as described above.

### *Haemonchus contortus*-induced NETs under acidic conditions

In order to verify whether NET formation occurs in abomasal acidic pH conditions, the cell culture medium (RPMI 1640) was adjusted to different acidic pH conditions: 2.0, 2.5, 3.0 and 3.5 prior to NET assays. For controls a pH of 7.0 was applied. Bovine PMN (*n* = 3, 5 × 10^5^) were seeded on poly-_L_-lysine pre-coated coverslips and exposed to *H. contortus* L_3_ (20 larvae/sample) in 300 μl RPMI medium (1 % PS, without phenol red) for 1 h at 37 °C. Thereafter NET formation was detected using a fluorescence microscope as previously described using an anti-histone H1, H2A/H2B, H3, H4 monoclonal antibody [mouse clone H11-4, 1:1000, 1 h, RT] jointly with a secondary antibody (Alexa Fluor® 488 goat anti-mouse IgG). Moreover, for staining extracellular DNA, Sytox Orange® (S-11368, Invitrogen, 1:1000, 15 min in the dark) was used.

### Statistical analysis

By using normal distribution of the data, co-culture/stimulation conditions were compared by one- or two-factorial analyses of variance (ANOVA) with repeated measures. Differences were regarded as significant at a level of *p* ≤ 0.05 and analysed by GraphPad Prism® programme.

## Results

### *Haemonchus contortus* L_3_ induce different types of NETs

**Fig. 1:**
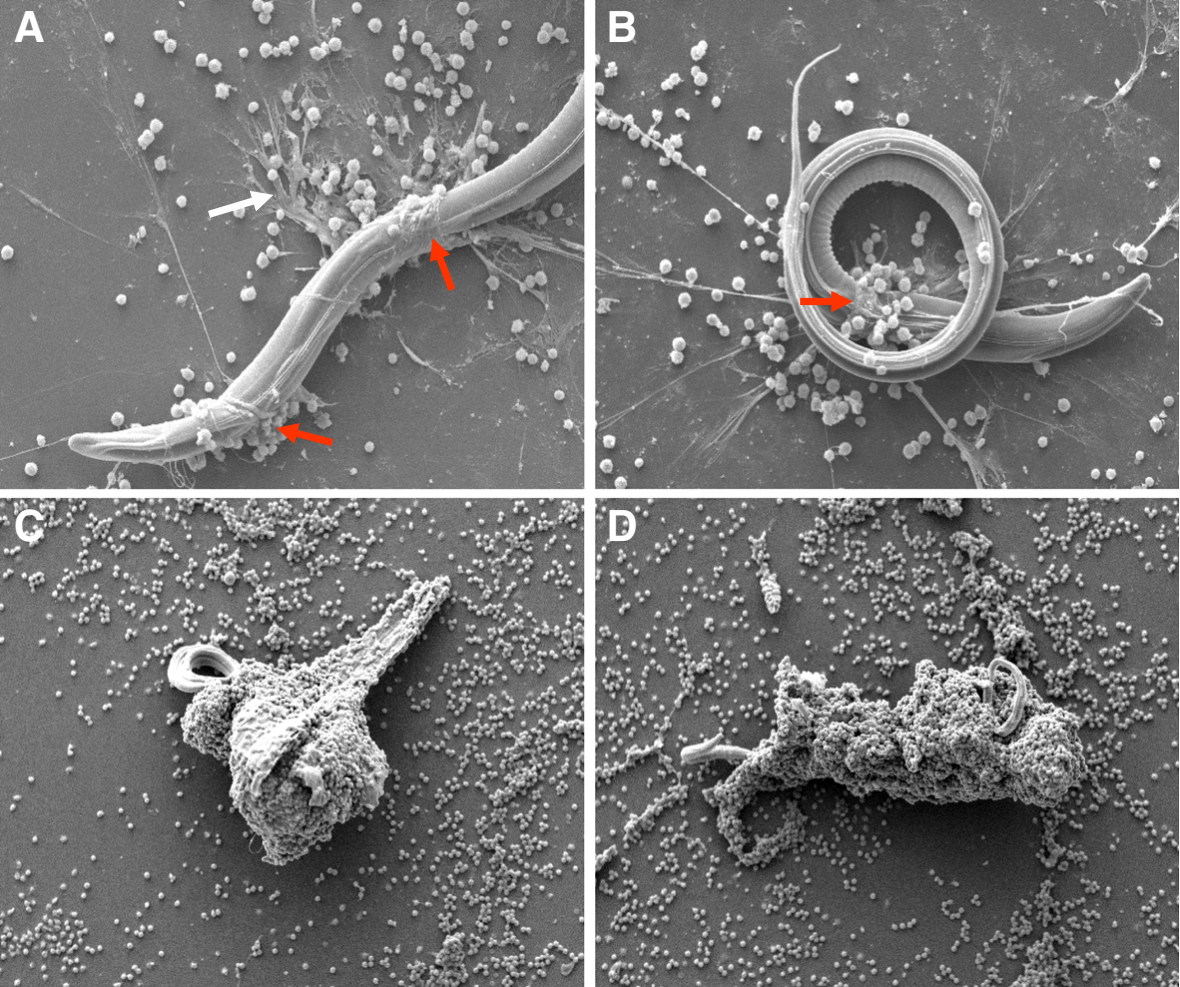
Co-culture of *Haemonchus contortus* larvae and bovine PMN triggers NET formation. Scanning electron microscopy (SEM) analysis revealed different forms of NET formation induced by *H. contortus* L_3_ in bovine PMN contributing to larval immobilization. **a**, **b**: *H. contortus* trigger spread NET formation (*spr*NET) **c**, **d**: *H. contortus* trigger aggregated NET formation (*agg*NET)

Scanning electron microscopy (SEM) analyses revealed that exposure of bovine PMN to ensheathed third-stage larvae of *H. contortus* triggers the formation of morphologically different NETs. Typically “diffuse” NETs (*diff*NETs) are composed of a complex of extracellular decondensed chromatin decorated with antimicrobial proteins with globular and compact form with a size of 25–28 nm diameter. Moreover, “spread” NETs (*spr*NETs) were observed consisting of smooth and elongated web-like structures of decondensed chromatin and antimicrobial proteins composed exclusively by thin fibers with a diameter of 15–17 nm (Fig. 1a, b). Furthermore, a marked presence of so-called “aggregated” NETs (*agg*NETs), according to [47] were displayed as large clusters of NET-like structures with a “ball of yarn”-like clumpy and massive appearance involving a high number of PMN. These conglomerates of NETs were composed by extracellular chromatin studded with phagocyte-granular proteins with sizes larger than 50 μm in diameter. *Spr*NETs and *agg*NET formation led to larval entrapment and, in some cases of *agg*NETs, to an almost entire coverage of the larvae (Fig. 1c, d). Overall, both forms of NETs, i.e. *spr*NET and *agg*NET, appeared capable of firmly ensnaring the larvae (see Additional files 1, 2: movies 1 and 2). Furthermore, we also observed a significant reduction in larval forward-motility due to an anchor-like effect of fine NET structures (*spr*NETs), which are connected to the *agg*NETs entrapping larvae and clearly hampering larval motility (see Additional files 1, 2: movies 1 and 2).

**Fig. 2:**
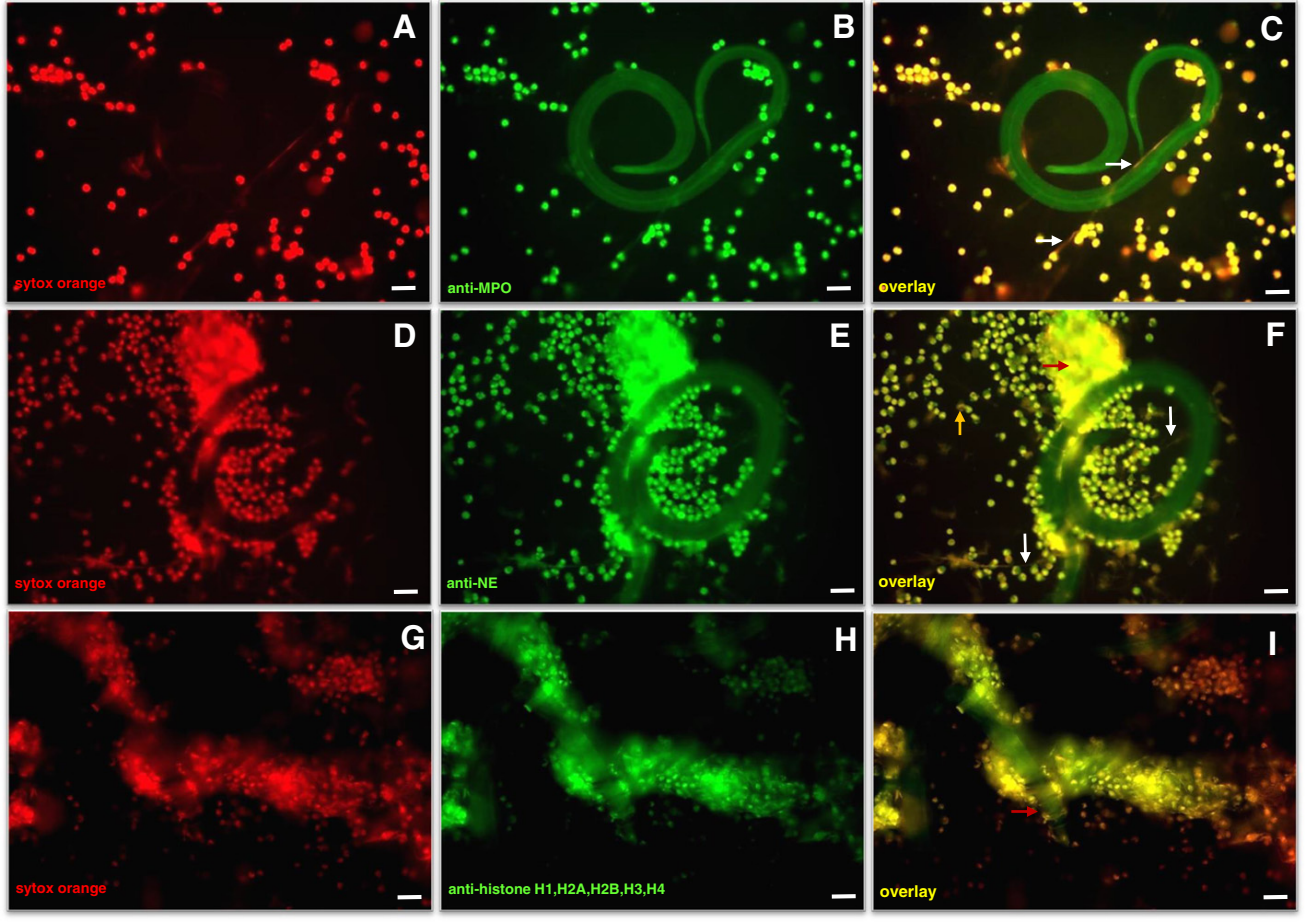
Co-localization of exracellular DNA with histones, MPO and NE in *H. contortus*-triggered NET structures. Co-cultures of bovine PMN with *H. contortus* L_3_ were fixed, permeabilized, stained for DNA using Sytox Orange and probed for histones using anti-histone H1, H2A/H2B, H3, H4 monoclonal antibodies, for MPO using anti-MPO and for NE using anti-NE antibodies. **a**, **d**, **g**: Extracellular DNA stained with Sytox Orange (red). **b**, **e**, **h**: MPO, NE and histones staining within NET structures (green), respectively. **c**, **f**, **i**: Overlay of NET-DNA with MPO, NE, and histones respectively. Note that distinct forms of NETs were observed in fluorescence microscopy: *spr*NET (**c**, **f** white arrows), *agg*NET (**f** red arrow) *diff*NET (**f** yellow arrow) and *clasp-like* NET (**i** red arrow). Photomicrographs represent exemplary images of 3 independent experiments

Fluorescence-based analyses confirmed the classical components of NET formation. As such, staining by Sytox Orange® (Fig. 2a, d, g) proved the DNA nature of extracellular NET-like structures being formed by PMN after *H. contortus* L3 exposure. In addition, MPO-, NE- and histone- (H1, H2A/H2B, H3 and H4) positive signals were detected in co-localization with DNA-positive NET structures (Fig. 2c, f, i). Interestingly, we also observed different forms of NETs, i.e., *spr*NETs (Fig. 2c, f, white arrows), *agg*NETs (Fig. 2f, red arrows), diffused NETs (*diff*NET, Fig. 2f, yellow arrow) and clasp-like NET formation (Fig. 2 i, red arrow) sticking mainly to the anterior part of the larvae, consistent with observations made by SEM analyses (see Fig. 1).

**Fig. 3:**
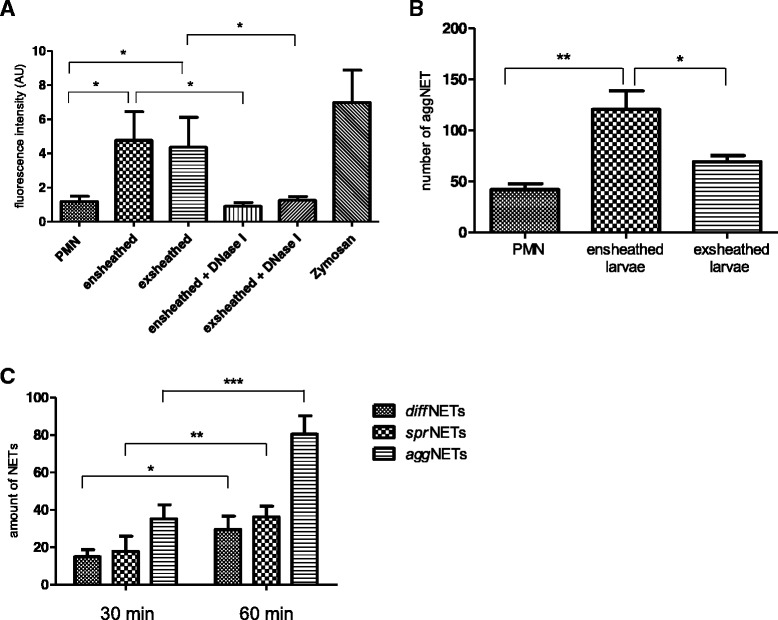
*Haemonchus contortus* L_3_ induce the formation of *agg*NET upon exposure to bovine PMN. **a**: Bovine PMN were confronted with ensheathed and exsheathed *H. contortus* L_3._ NET measurement was performed using PicoGreen-derived fluorescence intensities and DNase I was applied 15 min before the end of incubation to the co-culture of PMN with both ensheathed and exsheathed larvae. **b**: Bovine PMN were confronted with ensheathed and exsheathed *H. contortus* L_3_ and *agg*NET were counted using phase-contrast microscopy based on a size larger than 50 mm diameter. As negative control, PMN in plain medium were used. PMN exposed to zymosan (1 mg/ml) served as positive control. **c**: Bovine PMN (*n* = 3, 5 × 10^5^) were exposed to *H. contortus* L_3_ (20 larvae/sample) for 30 and 60 min at 37 °C. For the quantification of different types of NETs, fluorescence microscopy analysis was performed using an anti-histone H1, H2A/H2B, H3, H4 monoclonal antibody, [mouse clone H11-4, 1:1000, 1 h, RT], jointly with a secondary antibody (Alexa Fluor® 488 Goat Anti-Mouse IgG). Extracellular DNA was analysed via Sytox Orange® staining (S-11368, Invitrogen, 1:1000).

Quantitative analyses on total NET formation showed that ensheathed and exsheathed L_3_ significantly induced NETosis in bovine PMN (*p* ≤ 0.05, Fig. 3a). As expected, this reaction was significantly and almost entirely resolved by DNase I treatment in both, exsheathed and ensheathed larvae (larvae vs. DNase I: *p* ≤ 0.05, Fig. 3a). Furthermore, quantitative assessment of *agg*NETs revealed that exposure of PMN to ensheathed L_3_ significantly induced *agg*NET in bovine PMN when compared to negative controls (*p* ≤ 0.01, Fig. 3b). Comparative approaches revealed that ensheathed larvae triggered NETosis significantly more than exsheathed ones (*p* ≤ 0.05, Fig 3b), indicating that surface cuticle components are able to effectively trigger NETosis. Furthermore, analysis of the frequency of different NET types induced by *H. contortus* was performed for different time spans (30 and 60 min). As outcomes of this experiment, at both time points the three different types of NETs were observed to be significantly increased (*p* ≤ 0.001, Fig. 3c). Interestingly, at both time points predominantly *agg*NET were detected more frequently. Most probably, sprNET and diffNET are stages of the same process being *agg*NETs the latest stage. The difference of *diff*NET and *spr*NET formation along the two time points exhibited a significant increased (*p* ≤ 0.05 and *p* ≤ 0.01, respectively; Fig. 3c) showing overall a clear time-dependent pattern.

### *Haemonchus contortus* L_3_-triggered NETosis occurs irrespective of parasite viability or pH environmental conditions

**Fig. 4:**
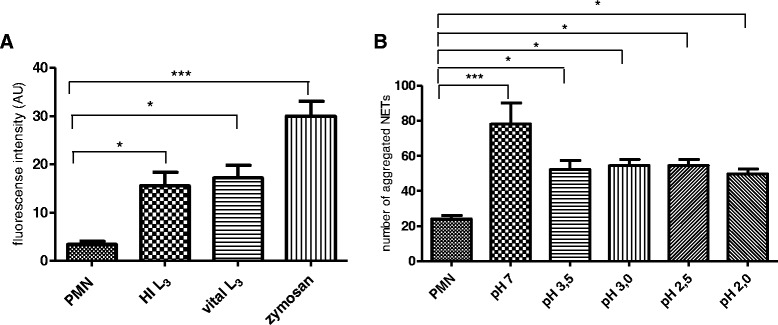
*H. contortus*-triggered NETosis is independent on the parasite integrity and low pH conditions. **a**: Bovine PMN were exposed to vital ensheathed larvae, heat-inactivated larvae or parasite-free controls. NET measurement was performed using PicoGreen-derived fluorescence intensities. **b**: Bovine PMN were exposed to *H. contortus* L_3_ at different pH conditions (pH 2.0–3.5) and NET measurement was performed using Sytox Orange®-derived fluorescence intensities with an anti-histone H1, H2A/H2B, H3, H4 monoclonal antibody, [mouse clone H11-4, 1:1000, 1 h, RT], jointly with a secondary antibody (Alexa Fluor® 488 Goat Anti-Mouse IgG). As positive control, stimulation of PMN by zymosan (1 mg/ml) was used. For negative control, PMN in plain medium were used

In order to analyse the role of *H. contortus* L_3_ viability and integrity in parasite-induced NETosis, either viable or heat-inactivated larvae were used for NET induction. The quantitative assessment of NETs revealed that both preparations equally induced NETosis (viable and heat-inactivated larvae vs. control: both *p* ≤ 0.05; Fig. 4a), i.e. the viability or integrity of larvae is irrelevant for NET induction.

Given that, following oral infection, ensheathed L_3_ of *H. contortus* reach the acidic environment of the abomasum and, after exsheathment, infect abomasal glands, we additionally tested whether NETosis also occurs in acidic conditions by testing different pH ranges (2.0, 2.5, 3.0 and 3.5) of the culture medium. Overall, even under low acidic conditions NETs were significantly formed in response to *H. contortus* L_3_ (pH 2.0 vs. control: *p* ≤ 0.05; pH 2.5 vs. control: *p* ≤ 0.05; pH 3 vs. control: *p* ≤ 0.05; pH 3.5 vs. control: *p* ≤ 0.05, Fig. 4b). However, at pH 7 a significantly stronger reaction was observed compared to the negative control (*p* ≤ 0.001) and lowering of the pH to 2.0 led to a reduction of NET formation of approximately 30 % when compared to pH 7 (Fig. 4b).

### NETs entrap *Haemonchus contortus* L_3_ in a time-dependent and non-lethal manner

**Fig. 5:**
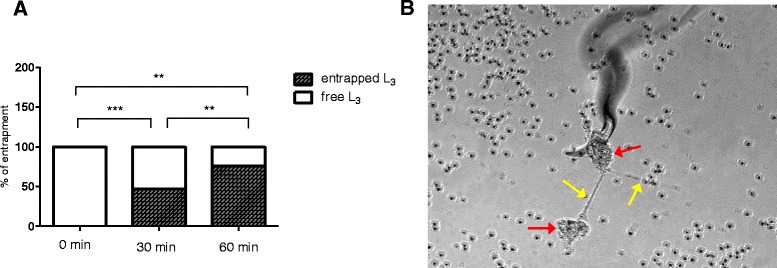
Entrapment of *H. contortus* L_3_ within NET structures is time-dependent. **a**: Bovine PMN were co-cultured with *H. contortus* L_3_. Entrapped larvae were counted microscopically under different time points. **b**: Phase contrast image showing the anterior part of a L_3_ being entrapped in NET structures. Red arrows: *agg*NET. Yellow arrows: anchor-like effect of fine NET structures mainly originating from *agg*NETs

In order to evaluate time dependency of *H. contortus*-mediated NET entrapment, bovine PMN were incubated with *H. contortus* for different time spans. As a result, significant differences were observed between 30 and 60 min of exposure (*p* ≤ 0.01, Fig. 5) leading to 46.9 % and 75.8 % entrapment of larvae, respectively, indicating that this process is time-dependent.

To elucidate whether *H. contortus* L_3_ may be killed by NETs, parasite survival was evaluated by microscopic measures (motility + trypan blue exclusion test) following long-term PMN exposure. Overall, *H. contortus* larvae appeared reduced in their motility since only 80 % of the larvae displayed active motility compared to 100 % in non-exposed control larvae (data not shown). However, trypan blue exclusion test neither revealed damage of the larval cuticle nor trypan blue up-take indicating that the larvae were still alive after exposure to NETs.

### *Haemonchus contortus*-triggered NET formation is NADPH oxidase and MPO-dependent

**Fig. 6:**
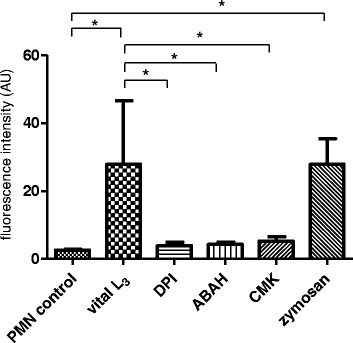
Blockage of *Haemonchus contortus* L_3_-triggered NETosis via inhibitors of NADPH oxidase, MPO, NE. NET inhibition assays were performed by pre-incubating PMN with inhibitors of NADPH oxidase (DPI, 10 μM), NE (CMK, 1 mM), MPO (ABAH, 100 μM) prior to *H. contortus* exposure. NET measurement was performed using PicoGreen-derived fluorescence intensities. For positive controls, zymosan treatment of PMN was used (1 mg/ml). PMN in plain medium served as negative control

To elucidate the role of different NET-associated molecules in *H. contortus* L_3_-triggered NETosis, inhibition experiments were performed using blockers of NADPH oxidase (DPI), MPO (ABAH) and NE (CMK). Pretreatments of PMN with DPI, CMK and ABAH resulted in a significant reduction of parasite-induced NET formation when compared with controls lacking inhibitors (*p* ≤ 0.05; Fig. 6), indicating a key role of NADPH oxidase, NE and MPO in *H. contortus* L_3_-mediated NETosis.

### *Haemonchus contortus* L_3_ induce eosinophil-derived ETs

**Fig. 7:**
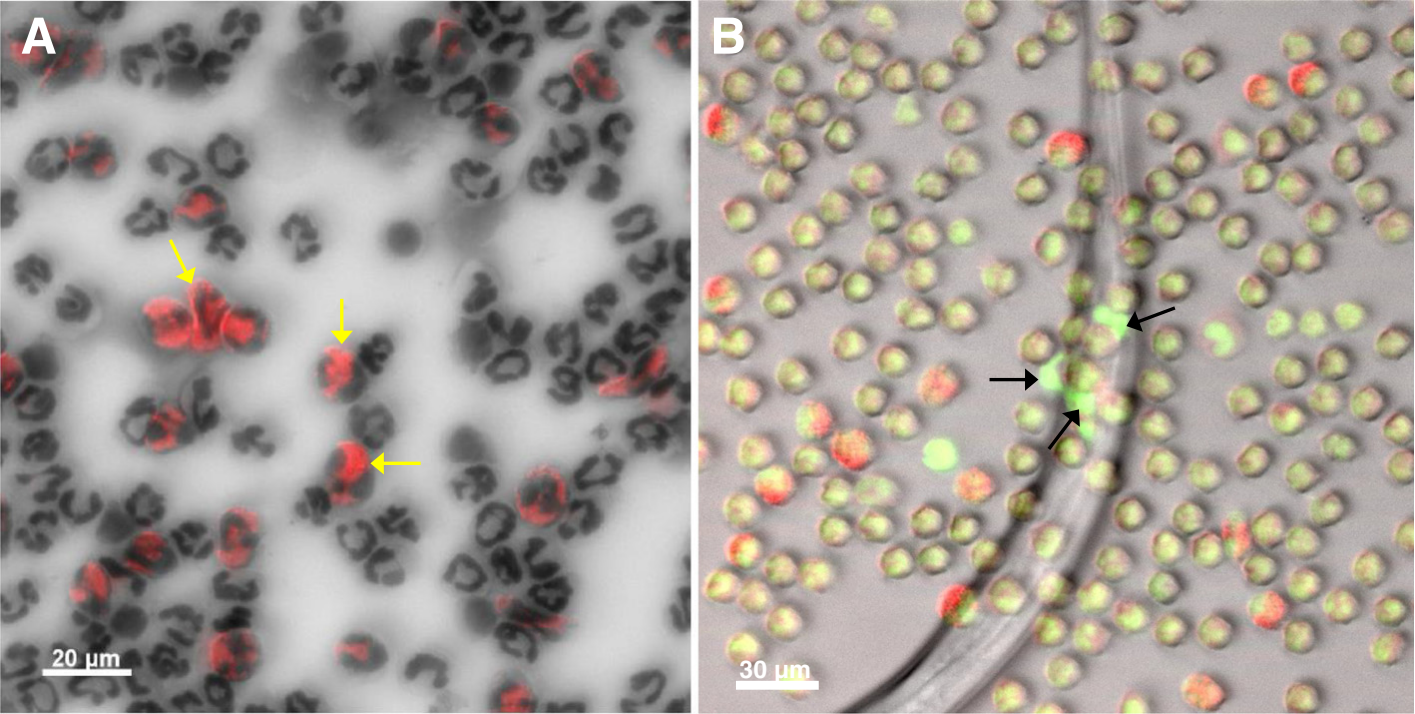
*Haemonchus contortus* L_3_ induce ETs in ovine eosinophils. **a**: Eosinophil population from ovine blood showing 30 % of enrichment as detected in Diff-Quick-stained cytospin preparations. **b**: Eosinophil-derived ETs being released towards an *H. contortus* L_3_ [see co-localization of eosin granules (red) with eosinophil-derived extracellular DNA (green)]

Ovine eosinophils were isolated at a purity of 30 % as demonstrated in Diff-Quick-stained cytospins (Fig. 7a). Given that the proportion of eosinophils in these preparations was not high enough to perform reliable quantitative assays on ETosis and further enrichment attempts failed, we made attempts to detect ET formation microscopically. Following exposure to *H. contortus* L_3_, several eosinophils (indicated by their red granules) were found in direct contact to the larvae (Fig. 7b). In addition, some of these clearly extruded PicoGreen®-positive DNA onto the larval surface indicating that these cells performed ETosis (Fig. 7b).

## Discussion

The present study delivers first evidence on the release of ETs as part of the early innate immune response of PMN and eosinophils to third stage larvae of the nematode *H. contortus*, an important and pathogenic species in the global veterinarian sector [6, 48–50]. NETs mainly consist of chromatin, nuclear histones (H1, H2A/H2B, H3, H4) and granular components, such as NE, MPO, lactoferrin and gelatinase [16, 51, 52]. We here confirmed the typical characteristics of NETs by co-localization experiments on extracellular DNA and histones, NE and MPO. Furthermore, functional inhibition experiments proved the relevance of NADPH oxidase, NE and MPO in *H. contortus*-induced NETosis since treatments with specific inhibitors significantly reduced parasite-triggered NET formation. This is in accordance to other reports on parasite-induced NET formation [30, 31, 39, 40, 45, 53].

During haemonchosis, infective *H. contortus* L_3_ must actively migrate through the lumen of the reticulum, the rumen and the abomasum in order to infect gastric glands of the latter organ. By performing this migration, *H. contortus* larvae become potential targets of leucocytes of the innate immune system, which are known to actively migrate into the intestinal lumen [54–57]. This encounter drives the initiation of innate immune reactions as previously demonstrated by O’Connell et al. [58] showing a CXCR2/IL-17 dependent neutrophil recruitment in response to nematode infections. Referring to the infection biology of *H. contortus*, it has to be considered that exsheathed larvae will reside within the abomasum under low pH conditions. However, the current data on pH-dependent parasite-triggered NET formation clearly indicate that NETosis also occurs in these acidic conditions.

So far, several reports exist on protozoan-induced NET formation [31, 35–40, 45, 53, 59] whilst only few data are available on metazoan-triggered NETosis (for overview see Hermosilla et al., [26]). As such, *S. japonicum* was recently identified as NET-inducer in vitro and in vivo [41]. Another report demonstrated the capability of the nematode *Strongyloides stercoralis* to trigger NETosis [15]. In agreement with the current study, a NET-based ensnarement of *S. stercoralis* larvae was also demonstrated [15].

As an interesting feature, observed in this study was the release of different types of NETs, i.e. of *diff*NETs, *spr*NETs and *agg*NETs upon contact with *H. contortus* larvae, with all of them promoting a time-dependent ensnarement of the larvae. Given that no parasite killing was observed even within a prolonged period of 24 h, the tight immobilization of larvae appears as the key mechanism of *H. contortus*-triggered NET formation. Therefore, we hypothesize that even though NETs themselves do not appear capable of killing *H. contortus* larvae, they may entrap larvae and prevent the active migration into the gastric glands of the abomasum and expose them to other leucocytes.

It is intriguing that parasite-mediated NET induction proved to be independent of the ensheathment status of the larvae, since both, ensheathed and exsheathed larvae equally triggered NET formation. So far, it is unclear how PMN recognize the larvae in terms of NETosis and which parasite-derived molecules are involved this process. However, the data on ex- and ensheathed larvae suggest that the molecules responsible are present on the cuticle and the surface of the larval body. In contrast, the fact that ensheathed larvae induce *agg*NETs more prominently than exsheathed ones also indicates the involvement of different parasite-derived triggers. However, PMN-derived sensing of the larvae may also be a matter of size since Branzk et al. [60] reported on the ability of PMN to distinguish between small and large sized pathogens and to selectively release NETs in response to large pathogens [60]. In addition, other physical properties of particles such as shape and rigidity can influence the response of phagocytes [61]. This would explain the induction of NET formation by large metazoan parasites such as *H. contortus* larvae, and makes biological sense as phagocytosis is probably ineffective against large multicellular pathogens. We hypothesize that a function of PMN-released *agg*NETs may be to prevent proper larval exsheathment in vivo. A blockage of larval exsheathment would abrogate parasite infections and thereby tremendously affect the outcome of disease. We also consider that ETs might serve to localize anthelmintic products of neutrophils and eosinophils in the near vicinity of parasites. As such, neurotoxin (eosinophil-derived neurotoxin or EDN) released by eosinophils would be more effective if localized near the parasite. The release of EDN may restrict motility of the larvae thereby preventing the shedding process and allowing adhering eosinophils to discharge their toxic granule content directly on the larval surface [20, 62]. This might serve to enhance the effects of these molecules impacting on larval motility. Moreover, parasite killing may be more likely to succeed if these anthelmintic molecules remain concentrated in the near vicinity of the parasite. Furthermore, ETosis is not a unique feature of PMN [63] but is also attributed to other immune cells, such as macrophages [64], mast cells [27, 65], monocytes [30, 31] and eosinophils [29, 66]. In accordance, we provide evidence on *H. contortus*-*induced eosinophil-derived ETs (EETs). Meanwhile, in vivo studies with the nematode Nippostrongylus brasiliensis* [62] demonstrate the formation of similar 'ET like entities' in presence of eosinophil-rich leucocytes and complement. In this model, larvae recovered from skin air pouches were aggregated into large clumps that included numerous leucocytes. Whilst many larvae eventually escaped from these aggregations, in mice with pre-existing eosinophilia, subsequent larval migration to the lungs was impaired [62]. This suggests that ET, leucocytes, and especially eosinophils, may be involved in damaging larvae in the very earliest stages of infection, such that the parasites are then more susceptible to entrapment and killing elsewhere in the pre-lung phases of migration through the host [62]. This may be of high relevance since eosinophils have been shown to adhere to *H. contortus* larvae both in vitro and in vivo and to efficiently kill these larvae [20]. Interestingly, Yousefi et al. [29] demonstrated that eosinophils release EETs of mitochondrial origin without undergoing cell death. However, whether *H. contortus-*triggered EETs also originating from mitochondria remains to be elucidated in the future. Nonetheless, our data strongly suggest EETs as an additional effector mechanism of eosinophils against *H. contortus.*

## Conclusion

Overall, we postulate that NETs and EETs may limit the establishment of *H. contortus* within the definitive host by immobilizing the larvae and hampering them from migration to the site of infection. NETs and EETs may further facilitate the exposure of entrapped large sized parasites to other immunocompetent cells, such as monocytes and macrophages exhibiting larvicidal effects. In consequence, *H. contortus*-mediated ET formation will also have an impact on the in vivo situation and influence the outcome of the disease.

## Additional file

Additional file 1:
**Parasite entrapment by** ***agg*** **NETs and the anchor-like effect of fine NET structures (** ***spr*** **NETs) derived from** ***agg*** **NETs.** (AVI 474248 kb) Additional file 2:
**Significant reduction in larval motility due to an anchor-like effect of fine NETs originated from** ***agg*** **NETs.** (AVI 398927 kb)
